# MicroRNA-126-3p is Downregulated in Human Kidneys in a Model of Reperfusion Injury

**DOI:** 10.1016/j.ekir.2020.09.035

**Published:** 2020-09-24

**Authors:** Nina P. Jordan, Michael L. Nicholson, Sarah A. Hosgood

**Affiliations:** 1 Department of Surgery, University of Cambridge, Addenbrooke's Hospital, Cambridge, UK

One of the main challenges of deceased donor kidney transplantation is to reduce rates of delayed graft function. However, added warm ischemic injury in kidneys from donation after circulatory death (DCD) donors and donor comorbidities such as increasing age, cardiovascular disease, diabetes, and hypertension are complicating factors that are associated with early graft dysfunction and reduced graft survival.^1^

One potential strategy to improve early graft function is to reduce the severity of ischemia reperfusion injury (IRI) in the early transplantation phase. IRI involves interlinked events that lead to the impairment of microvasculature circulation, upregulation of inflammatory and pro-fibrotic mediators, and modulation of procoagulation pathways.^2^ Endothelial cell damage and dysfunction are central in the role of renal IRI and, in the long-term, sustained injury can lead to the early development of fibrosis to cause graft loss.^2^ Targeting endothelial cell dysfunction during IRI could improve graft function.

MicroRNAs (miRNAs) are small non-coding RNAs that are involved in the regulation of gene expression.^3^ Their detection has been associated with many different disease states and they are considered to be useful biomarkers and targets for the development of therapeutic agents.^3^ MiR-126-3p is highly enriched in endothelial cells and participates in the regulation of angiogenesis and vascular integrity.^4^ In a recent study, the downregulation of miR-126-3p was among a panel of miRNAs associated with acute kidney injury in a small cohort of patients.^5^ Furthermore, reduced expression of miR-126-3p has also been associated with chronic kidney disease.^6^ There is no evidence describing a role for miR-126-3p in renal IRI. The aim of this pilot study was to establish a model of IRI using human kidneys and examine the expression of miR-126-3p.

## Results

### Hemodynamics and Renal Function

Four DCD kidneys that were declined for transplantation and offered for research were reperfused on an *ex vivo* circuit with oxygenated compatible fresh whole blood for 4 hours at 36 ± 1 °C. The donors had a range of comorbidities and the kidneys were declined for transplantation due to a variety of reasons (Table 1). The mean donor age was 56 ± 7 years, warm ischemic time was 15 ± 1 minutes, and cold ischemic time was 22.3 ± 4.6 hours.

**Table 1:** Kidney characteristics with donor type, cause of death, age, sex, past medical history, reason for decline for transplantation, warm ischemia time and cold ischemia time

Characteristics	Kidney 1	Kidney 2	Kidney 3	Kidney 4
Donor type	DCD	DCD	DCD	DCD
Cause of death	Hypoxic brain injury	Intracranial hemorrhage	Hypoxic brain injury	Intracranial hemorrhage
Donor age, yr	55	47	63	60
Donor sex	M	M	M	M
Past medical history	Out of hospital cardiac arrest 20 min	Liver and cardiovascular disease	Type II diabetes	Hypertension, cardiovascular disease, pre-diabetic
Reason for decline	Raised terminal creatinine (138 μmol/l)	Prolonged period of hypotension after withdrawal of treatment	Renal cell carcinoma on other kidney	Damage to the ureter and polar artery
Warm ischemic time, min	15	17	15	14
Cold ischemia time, h	18.6	20.4	21.1	29.0

DCD, donated after circulatory death; M, male.

**Figure 1:**
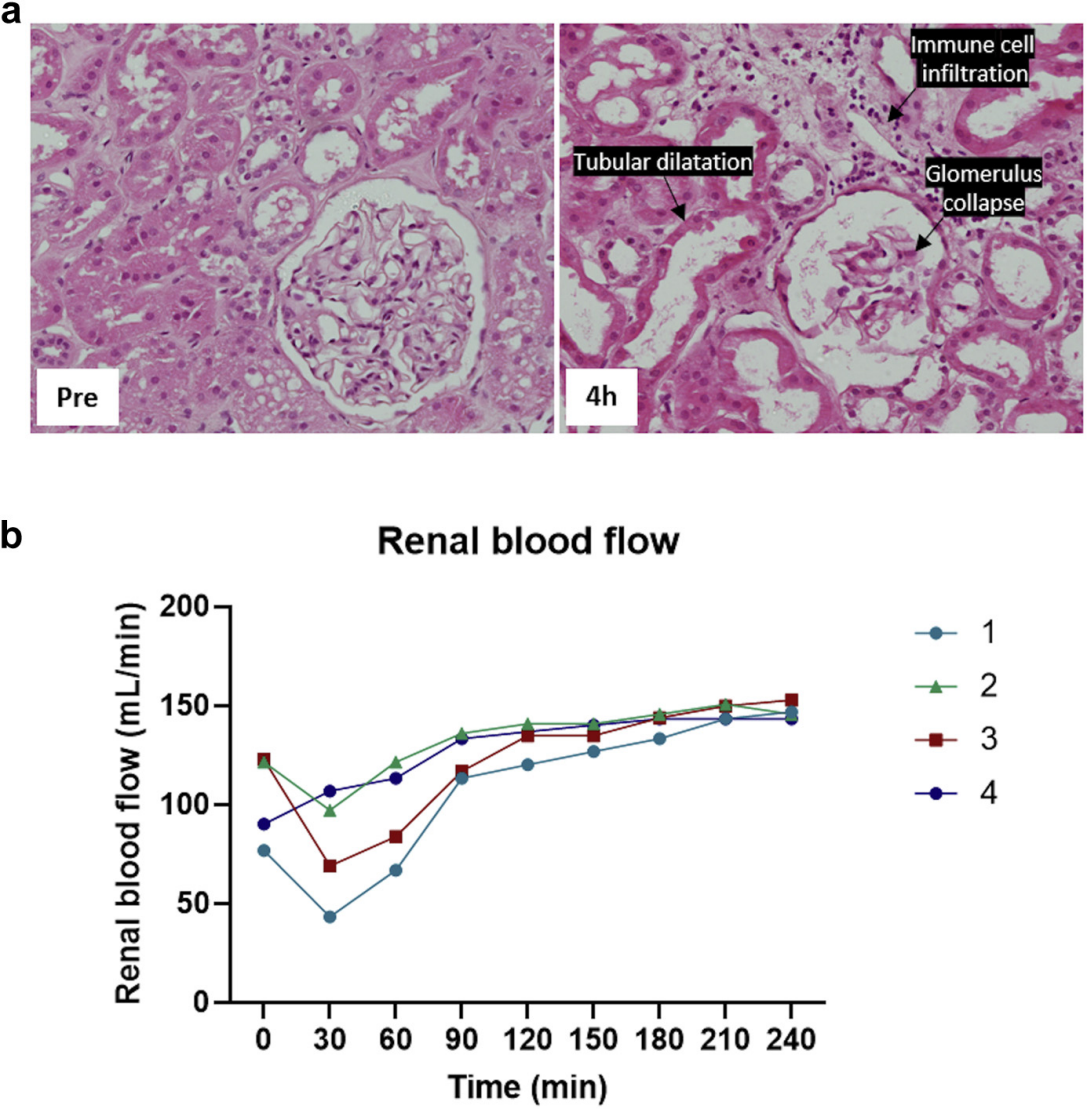
(a) Hematoxylin and eosin staining shows histopathologic changes after 4 hours of reperfusion. (b) Renal blood flow was measured every 30 minutes during the 4 hours of reperfusion.

In three kidneys, renal blood flow declined over the first 30 minutes of reperfusion before recovering to a baseline level after 4 hours (Figure 1b). The amount of urine produced varied significantly, ranging from a total of 10 to 121 ml over 4 hours of reperfusion. All kidneys showed significant renal dysfunction with low levels of creatinine clearance (Table 2).

**Table 2:** Mean ± SD levels of CrCl, total urine output, and histology evaluation after 4 hours of *ex vivo* reperfusion with whole blood

Characteristics	Kidney 1	Kidney 2	Kidney 3	Kidney 4
CrCl, ml/min per 100 g	0.02 ± 0.01	0.03 ± 0.01	0.40 ± 0.32	0.30 ± 0.14
Total urine output, ml	9.5	12.0	116.0	121.0
Histology^a^	Moderate	Severe	Moderate	Severe

CrCl, creatinine clearance.

a Histologic evaluation sections were graded mild, moderate, or severe for acute tubular injury.

### Inflammation, Endothelial Damage, Histologic Evaluation, and miR-126-3p Expression

**Figure 2:**
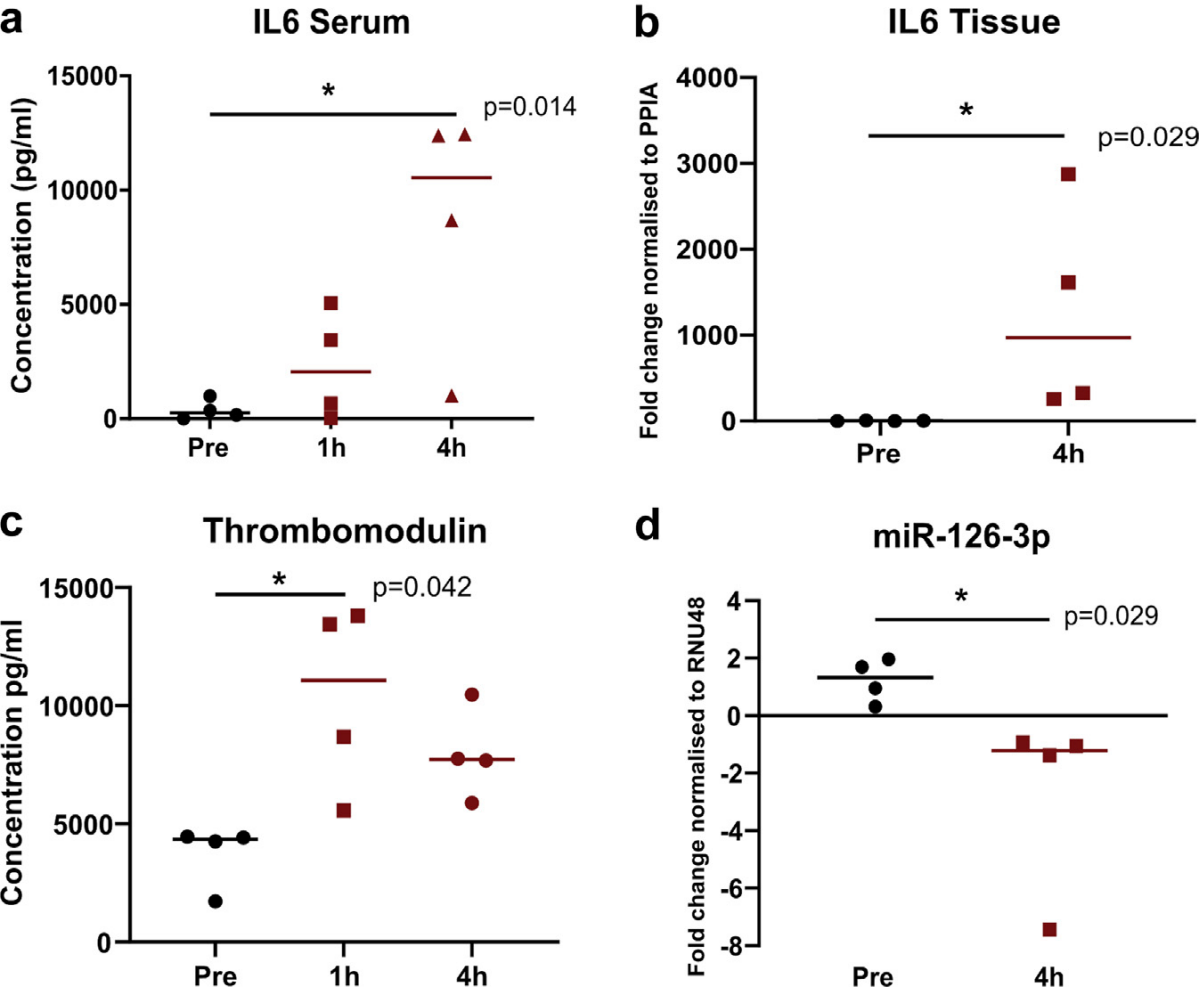
MiR-126-3p expression in *ex vivo* reperfusion human kidney model. Levels of IL6 in the perfusate (a) and in the tissue (b) measured before reperfusion, 1 and 4 hours after reperfusion. (c) Soluble thrombomodulin expression in the perfusate during reperfusion. (d) Expression of miR-126-3p in tissue pre-reperfusion and 4 hours after reperfusion (∗*P* < 0.05).

Levels of interleukin 6 (IL-6) measured in the circulating perfusate were variable. However, there was a significant increase in levels at 1 and at 4 hours of reperfusion compared to baseline (*P* = 0.014) (Figure 2a). In addition, gene expression of IL-6 was significantly upregulated in the tissue after 4 hours of reperfusion (*P* = 0.029) (Figure 2b).

Levels of soluble thrombomodulin in the perfusate were significantly increased at 1 and 4 hours of reperfusion in all kidneys compared to baseline (*P* = 0.042) (Figure 2c).

Histopathologic evaluation showed acute tubular injury and ischemic changes with glomerular collapse, tubular vacuolation, tubular dilatation, and infiltration of immune cells after 4 hours reperfusion in all kidneys (Figure 1a, Table 2). Kidneys 2 and 4 showed severe acute tubular injury and the others moderate.

The expression of miR-126-3p was significantly downregulated in all kidneys after 4 hours of reperfusion compared to baseline (*P* = 0.029) (Figure 2d).

## Discussion

This study showed that during reperfusion miR-126-3p was significantly downregulated in human DCD kidneys.

The upregulation of pro-inflammatory cytokines, promotion of cell adhesion and infiltration, vasoconstriction, and increased immunogenicity of the kidney due to endothelial cell injury are complicating factors that cause early graft dysfunction. MiR-126-3p is an endothelial-specific miRNA with an important role in vascular integrity. It is highly expressed in endothelial cells but not in populations of leucocyte cell lines or vascular smooth muscle cells.^7^ However, it has been detected at a low level in mesangial cells and podocytes.^8^ In addition to the benefits of miR-126-3p in vascular remodelling, miR-126-3p may also have an important role in renal IRI. MiR-126-3p in serum has been identified as a biomarker of acute kidney injury and low levels have been associated with chronic graft rejection.^5^^,^^6^ Low levels of expression were also observed in biopsy specimens from patients with acute kidney rejection.^9^ The enhancement of miR-126-3p expression with the therapeutic agent paeonol has been shown to inhibit monocyte adhesion and block the activation of the P12K/Akt/NF-κB signalling pathway, an important mediator of IRI.S2

In this present study, the downregulation of miR-126-3p after 4 hours of reperfusion was associated with reduced kidney function, tubular injury, endothelial cell damage measured by high circulating levels of thrombomodulin, an endothelial transmembrane protein involved in coagulation signalling through the protein C pathway, and high levels of the pro-inflammatory cytokine IL-6 in all kidneys. Kidney 4, which is from a donor with a history of significant cardiovascular disease, had the most severe acute tubular injury, the highest levels of thrombomodulin and IL-6, and the greatest downregulation of miR-126-3p. Increased expression of IL-6 promotes neutrophil infiltration, exacerbates renal injury, and its sustained expression is linked to the development of fibrosis. Recent evidence suggests that IL-6 can also modulate the expression of miR-126-3p. In endothelial cells, IL-6 downregulated the expression of miR-126-3p and increased levels of intercellular adhesion molecule-1.S3

Targeting the expression of miRNAs is a potential new treatment for endothelial dysfunction in IRI. A number of different techniques are being investigated and include the use of gene therapy vectors and chemically modified RNAs or miRNAs chemically bound to nanoparticles to modify expression.^3^ The CRISPR/Cas 9 system can also be used to edit the miRNA sequence to enhance function.^3^

This preliminary study is the first to assess miR-126-3p in human kidneys undergoing a simulated period of reperfusion. Using compatible fresh whole blood the model allowed a detailed assessment of vascular integrity, acute graft function, and mechanistic effects of IRI in an isolated perfused kidney.

The study is limited by the inclusion of only four DCD kidneys and the inevitably narrow range of results. We consider that although the kidneys had been declined for transplantation they were of acceptable quality. The quality of the renal parenchyma was unaffected in two kidneys declined due to a donor malignancy and one due to a damaged ureter. These were essentially normal kidneys for the donor age group with the inevitable addition of ischemic injury. In the fourth kidney the only quality issue was a minimally elevated terminal serum creatinine level (138 μmol/l — against an upper limit of normal of 130 μmol/l). Histologic evaluation after reperfusion showed moderate or severe acute tubular injury in each of the kidneys. This is unsurprising in DCD kidneys after transplantation and consistent with the evolution of acute tubular necrosis. A select group of markers was used to determine the level of inflammation and endothelial cell damage. MiR-126-3p is known to suppress inflammation and reduce reactive oxygen species when stimulated by high glucose levels.S4 Therefore, future work should also consider the measurement of oxidative damage, complement activity and a broader range of inflammatory and immune mediators. This study provides salient preliminary data on the downregulation of miR-126-3p in a model of reperfusion that will inform further research in renal IRI.

## Disclosure

All authors declared no competing interests.
