# Supplementary material for: MicroRNA-126-3p is Downregulated in Human Kidneys in a Model of Reperfusion Injury
Source: Kidney Int Rep. 2020 Sep 24;5(12):2357–60. doi: 10.1016/j.ekir.2020.09.035 (PMC7710817; doi:10.1016/j.ekir.2020.09.035)
Supplement: Supplementary File PDF [file mmc1.pdf]

Supplemental Material

Material and Methods

Ethics

Four human DCD kidneys that are deemed unsuitable for transplantation and declined by transplant centres allocated from the national kidney service were used for this research project. Consent for research was obtained from the donor families by specialist nurses in organ donation. The research study was approved by the National ethics committee (NRES: 15/NE/0408). Fresh units of compatible whole blood were obtained by Cambridge Bioscience (Cambridgeshire, UK). Consent was granted from each of the blood donors.

Ex- vivo reperfusion

Kidneys were perfused with a unit of fresh whole blood mixed with Ringer's solution. After transportation on ice and arrival at the transplant laboratory the kidneys underwent a period of 4h reperfusion on an *ex-vivo* kidney perfusion system.[S1] Mannitol 10% 15ml (Baxter Healthcare), and heparin 1000iu/ml 3ml (CP Pharmaceuticals, Wrexham, UK) were added to the perfusate. Sodium bicarbonate 8.4% (Fresenius Kabi, Cheshire, UK) was added to normalise the pH. A nutrient solution (Synthamin 17**,** 10% Amino Acid Intravenous Infusion) with sodium bicarbonate 15ml 8.4% and insulin 100 IU (Novo Nordisk, Denmark) added was infused into the circuit at a rate of 20ml/h. Glucose 5% (Baxter Healthcare) was infused into the system at a rate of 5ml/h. Ringer's solution was used to replace urine output ml for ml. The perfusate was oxygenated with 95% oxygen 5% carbon dioxide and circulated through the kidney via the renal artery at a temperature of 36°C using a pump at 1460rpm to maintain a mean pressure of 85mmHg. The perfusate drained from the renal vein back into the venous reservoir and recirculated. Creatinine (1000μmol/L) was added to the perfusate.

Sample collection

During reperfusion, renal blood flow was recorded continually. Cortical biopsies were collected before and after 4h of reperfusion. Sections were frozen into liquid nitrogen for the evaluation of levels of miRNA-126-3p and IL-6. Sections were also taken for histological evaluation after fixation in formalin 10% and paraffin embedded. Samples of perfusate and urine were collected before and each hour during reperfusion. Samples were screened for routine haematology and biochemical analysis. Creatinine clearance was calculated each hour (urine creatinine x urine output (ml)/plasma creatinine).

Protein analysis

The pro-inflammatory cytokine IL-6 and the soluble thrombomodulin expression were assessed in perfusate samples by ELISA according to manufacturer's instruction (R&D, Minneapolis, USA) (Abcam, Cambridge , UK).

Real-Time Polymerase Chain Reaction

Total RNA including small RNAs was isolated from lysed and homogenized frozen tissue using a miRNeasy extraction kit (Qiagen, Hilden, Germany). cDNA synthesis from miRNAs and messenger RNA was performed using Taqman microRNA reverse transcription kit (Thermofisher, Waltham, USA) and high capacity cDNA reverse transcription kit respectively. Real-time polymerase chain reactions were carried out with Taqman assay probes or with Taqman miRNA assay (Thermofisher). (hsa-miR126-3p : UCGUACCGUGAGUAAUAAUGCG ; RNU48: GATGACCCCAGGTAACTCTGAGTGTGTCGCTGATGCCATCACCGCAGCGCTCTGACC)

Histology evaluation

Sections were graded mild, moderate or severe for acute tubular injury.

References

S1. Hosgood SA, Nicholson ML. An assessment of urinary biomarkers in a series of declined human kidneys measured during ex vivo normothermic kidney perfusion. Transplantation. 2017;101(9):2120-2125. doi:10.1097/TP.0000000000001504.

S2. Yuan X, Chen J, Dai M. Paeonol promotes microRNA-126 expression to inhibit monocyte adhesion to ox-LDL-injured vascular endothelial cells and block the activation of the PI3K/Akt/NF-κB pathway. Int J Mol Med. 2016;38(6):1871-1878. doi:10.3892/ijmm.2016.2778.

S3. Ohta M, Kihara T, Toriuchi K, et al. IL-6 promotes cell adhesion in human endothelial cells via microRNA-126–3p suppression. Exp Cell Res. 2020;393(2):112094. doi:10.1016/j.yexcr.2020.112094.

S4. Tang S tao, Wang F, Shao M, Wang Y, Zhu H qing. MicroRNA-126 suppresses inflammation in endothelial cells under hyperglycemic condition by targeting HMGB1. Vascul Pharmacol. 2017;88:48-55. doi:10.1016/j.vph.2016.12.002.
